# Three Distinct Two-Component Systems Are Involved in Resistance to the Class I Bacteriocins, Nukacin ISK-1 and Nisin A, in *Staphylococcus aureus*

**DOI:** 10.1371/journal.pone.0069455

**Published:** 2013-07-22

**Authors:** Miki Kawada-Matsuo, Yuuma Yoshida, Takeshi Zendo, Junichi Nagao, Yuichi Oogai, Yasunori Nakamura, Kenji Sonomoto, Norifumi Nakamura, Hitoshi Komatsuzawa

**Affiliations:** 1 Department of Oral Microbiology, Kagoshima University Graduate School of Medical and Dental Sciences, Kagoshima, Japan; 2 Department of Oral and Maxillofacial Surgery, Kagoshima University Graduate School of Medical and Dental Sciences, Kagoshima, Japan; 3 Laboratory of Microbial Technology, Division of Applied Molecular Microbiology and Biomass Chemistry, Department of Bioscience and Biotechnology, Faculty of Agriculture, Graduate School, Kyushu University, Fukuoka, Japan; 4 Department of Functional Bioscience, Section of Infection Biology, Fukuoka Dental College, Fukuoka, Japan; National Institutes of Health, United States of America

## Abstract

*Staphylococcus aureus* uses two-component systems (TCSs) to adapt to stressful environmental conditions. To colonize a host, *S. aureus* must resist bacteriocins produced by commensal bacteria. In a comprehensive analysis using individual TCS inactivation mutants, the inactivation of two TCSs, *graRS* and *braRS*, significantly increased the susceptibility to the class I bacteriocins, nukacin ISK-1 and nisin A, and inactivation of *vraSR* slightly increased the susceptibility to nukacin ISK-1. In addition, two ABC transporters (BraAB and VraDE) regulated by BraRS and one transporter (VraFG) regulated by GraRS were associated with resistance to nukacin ISK-1 and nisin A. We investigated the role of these three TCSs of *S. aureus* in co-culture with *S. warneri*, which produces nukacin ISK-1, and *Lactococcus lactis*, which produces nisin A. When co-cultured with *S. warneri* or *L. lactis*, the *braRS* mutant showed a significant decrease in its population compared with the wild-type, whereas the *graRS* and *vraSR* mutants showed slight decreases. Expression of *vraDE* was elevated significantly in *S. aureus* co-cultured with nisin A/nukacin ISK-1-producing strains. These results suggest that three distinct TCSs are involved in the resistance to nisin A and nukacin ISK-1. Additionally, *braRS* and its related transporters played a central role in *S. aureus* survival in co-culture with the strains producing nisin A and nukacin ISK-1.

## Introduction

Many bacteria produce antibacterial agents, called bacteriocins, which interfere with other bacteria in the bacterial community [1]. Bacteriocins are peptides or proteins that are ribosomally synthesized and show antimicrobial activity, mostly against bacterial species that are closely related to the producers [2]. In gram-positive bacteria, bacteriocins are classified into two major types, classes I and II bacteriocins [3], [4], [5]. Class I bacteriocins (<5-kDa peptides) are called lantibiotics because they contain the unusual amino acids, lantionine and methyllanthionine, which are posttranslationally modified, whereas class II bacteriocins contain unmodified amino acids. Lantibiotics are further divided into two types (types A and B) [6]. Type A lantibiotics include two subtypes, type A(I) such as nisin A and type A(II) such as nukacin ISK-1. The mode of action of lantibiotics, especially nisin A, has been well characterized [7], [8]. Nisin A exhibits pore-forming activity and the inhibition of cell wall biosynthesis. The docking molecule of nisin A is lipid II, which is a membrane component consisting of one GlcNAc–MurNAc pentapeptide subunit linked to a polyisoprenoid and is associated with peptidoglycan biosynthesis in the membrane [9]. Nukacin ISK-1 also binds to lipid II [10]. However, the mode of action of nukacin ISK-1 is not pore-forming, but the inhibition of cell wall synthesis causes a bacteriostatic effect [10], [11]. These bacteriocins are considered to affect other bacterial populations. In addition, some bacteriocins, such as nisin A, are used as preservatives for foods and other surfaces [1].

*Staphylococcus aureus* is a major pathogen in humans that can cause a variety of suppurative diseases, food poisoning, and toxic shock syndrome [12], [13], [14]. Furthermore, clinically isolated strains, particularly methicillin-resistant *S. aureus* (MRSA), exhibit multiple antibiotic resistances [15], [16], resulting in serious problems with regard to therapy against *S. aureus* infectious diseases. Several two-component systems (TCSs) such as *vraSR*, *agrCA*, and *graRS/apsRS*, were recently reported as being associated with susceptibility to antibacterial agents [17], [18], [19], [20]. A TCS, which is thought to function as a monitor and adapt to specific environmental conditions [21], is a prokaryote-specific signal transduction system that contains a sensor that encodes a sensory histidine-kinase and a regulator that encodes a cognate response regulator (RR) [22]. Recently, we and others identified one TCS, called BceRS [23], BraRS [24] or NsaRS [25], which affects susceptibility to bacitracin. In addition, this TCS is associated with resistance to nisin A [24], [25], [26]. Because this TCS was designated separately by three different groups, we used the name BraRS in this study because Hiron *et al* well characterized this TCS and the name (bacitracin resistance associated) is representative of its characteristics [24]. These findings suggest that *S. aureus* has several systems for resisting bacteriocins. Given that *S. aureus* is a commensal bacterium in the nasal cavity, skin, and intestine, this organism is faced with many other bacterial species, including other staphylococci such as *S. epidermidis* and *S. warneri* [27], [28]; thus it is considered that *S. aureus* must resist bacteriocins to survive when it co-exists with bacteriocin-producing bacteria. Herein, we investigated the association of TCSs with susceptibility to the class I bacteriocin, nukacin ISK-1.

## Materials and Methods

### Bacterial Strains and Growth Conditions

The bacterial strains used in this study are listed in Tables 1 and 2. *S. aureus* inactivation mutants were constructed previously [23]. *S. aureus* and *S. warneri* were grown in trypticase soy broth (TSB; Becton Dickinson Microbiology Systems, Cockeysville, MD, USA) at 37°C. *Escherichia coli* XL-II was grown in Luria-Bertani (LB) broth at 37°C. *Lactococcus lactis* was grown in De Man, Rogosa, Sharpe (MRS) broth (Becton Dickinson Microbiology Systems) at 37°C. Tetracycline (TC; 5 µg/ml) or chloramphenicol (CP; 10 µg/ml) was added to *S. aureus* when necessary.

**Table 1:** Bacterial strains.

Strain	InactivatedGene ID^1^	Gene Name	Characteristics	Reference
*Staphylococcus aureus*				
MW2	–	–	Clinical strain, methicillin-resistant (*mecA*+) *S. aureus*	18
MM30	–	–	MW2 harboring pCL8, CP^r2^	This study
FK61	MW0198-99	unassigned	MW0199:: pCL52.1 in MW2, TC^r3^	18
FK62	MW0236-37	*lytSR*	*lytS*:: pCL52.1 in MW2, TC^r^	18
FK64	MW0621-22	*graRS/apsRS*	*graR*:: pCL52.1 in MW2, TC^r^	18
FK65	MW0667-68	*saeRS*	*saeR*:: pCL52.1 in MW2, TC^r^	18
FK66	MW1208-09	unassigned	MW1208:: pCL52.1 in MW2, TC^r^	18
FK67	MW1304-05	*arlRS*	*arlR*:: pCL52.1 in MW2, TC^r^	18
FK68	MW1445-46	*srrAB*	*srr*A: pCL52.1 in MW2, TC^r^	18
FK69	MW1636-37	*phoPQ*	*phoP*: pCL52.1 in MW2, TC^r^	18
FK71	MW1789-90	unassigned	MW1790:: pCL52.1 in MW2, TC^r^	18
FK72	MW1824-25	*vraSR*	*vraS*:: pCL52.1 in MW2, TC^r^	18
FK73	MW1962-63	*agrCA*	*agrC*:: pCL52.1 in MW2, TC^r^	18
FK74	MW2002-03	*kdpDE*	*kdpD*: pCL52.1 in MW2, TC^r^	18
FK75	MW2282-83	*hssRS*	*hssR*:: pCL52.1 in MW2, TC^r^	18
FK76	MW2313-14	*nreBC*	*nreB*:: pCL52.1 in MW2, TC^r^	18
FK77	MW2544-45	*braRS*	*braR*:: pCL52.1 in MW2, TC^r^	18
MM08	MW2544	*braS*	*braS*:: pCL52.1 in MW2, TC^r^	23
MM09	MW2544	*braS*	pMM09(*braS*) in MM08, TC^r^, CP^r^	23
MM01	MW0623-24	*vraFG*	*vraF*:: pCL52.1 in MW2, TC^r^	This study
MM17	MW0623-24	*vraFG*	pMM17(*vraFG*) in MM01, TC^r^, CP^r^	This study
MM02	MW2543-42	*braAB*	*braA*:: pCL52.1 in MW2, TC^r^	23
MM07	MW2542	*braB*	*braB*:: pCL52.1 in MW2, TC^r^	23
MM10	MW2542	*braB*	pMM10(*braB*) in MM07, TC^r^, CP^r^	23
MM03	MW2620-21	*vraDE*	*vraD*:: pCL52.1 in MW2, TC^r^	23
MM11	MW2620-21	*vraDE*	pMM11(*vraDE*) in MM03, TC^r^, CP^r^	23
MM12	MW0621-21	*graRS/apsRS*	pMM12(*graR*) in FK64, TC^r^, CP^r^	This study
MM31	MW0621-22	*graRS/apsRS*	pMM11(*vraDE*) in FK64, TC^r^, CP^r^	This study
MM231	MW1824-25	*vraSR*	pMM231(*vraSR*) in FK72, TC^r^, CP^r^	This study
*Escherichia coli*				
XL-II	–	*–*	*endA1 supE44 thi-1 hsdR17 recA1 gyrA96 relA1* *lac* [F′ *proAB lacI*q*ZΔM15* Tn*10* (Tet^r^) Amy Cam^r^]	Stratagene
mm01	MW0623-24	*vraFG*	pMM01/*E. coli* XL-II for *vraF* inactivation, Amp^r4^	
mm17	MW0623-24	*vraFG*	pMM17/*E. coli* XL-II for *vraFG* complementation, Amp^r^	This study
mm12	MW0621-21	*graRS/apsRS*	pMM12/*E. coli* XL-II for *graR* complementation, Amp^r^	This study
mm31	MW0621-22	*vraDE*	pMM31/*E. coli* XL-II for *vraDE* complementation, Amp^r^	This study
mm231	MW1824-25	*vraSR*	pMM231/*E. coli* XL-II for *vraSR* complementation, Amp^r^	This study

1 Gene ID in *S. aureus* MW2.

2 Chloramphenicol resistance.

3 Tetracycline resistance.

4 Ampicillin resistance.

**Table 2:** Bacteriocin-producing and non-producing strains used in this study.

Strain	Bacrteriocin	Reference
*Lactococcus lactis* ATCC 11454	Nisin A (class I)	50
*Lactococcus lactis* NZ9000	Nisin A non-producing	10
*Staphylococcus warneri* ISK-1	Nukacin ISK-1 (class I)	31
*Staphylococcus warneri* ISK-1^-^	pPI-1 cured strain, nukacin ISK-1 non-producing	51

### Evaluation of Bacteriocin Susceptibility

Two methods (the minimum inhibitory concentration [MIC] and direct methods) were used to evaluate susceptibility to bacteriocins. MICs of nisin A, nukacin ISK-1, and bacitracin were determined by micro-dilution method as described previously [29]. Nisin A [30] and nukacin ISK-1 [31] were purified as described elsewhere. MICs were determined after 10 h of incubation. Three independent experiments were performed.

In the direct method, modified from a previous method [32], 2 µl of an overnight culture of *S. warneri* and *L. lactis* were spotted on an MRS agar plate. After overnight incubation at 37°C, 5 ml of pre-warmed TSB soft agar (0.75%) containing wild-type *S. aureus* or the mutants at 10^6^ cells/ml was poured over the TSB agar plate. Plates were incubated for 20 h at 37°C. We confirmed that the diameter of the producing colony was uniformly 7 mm among all strains. The diameter of the inhibition zones surrounding bacteriocin-producing strains was measured in three directions. Three independent experiments were performed for the direct method, and the average result of the three experiments was calculated. Statistical analysis was performed with Dunnett’s method.

### Effect of Nukacin ISK-1 and Nisin A on the Expression of TCSs and Transporters

A small portion (10^8^ cells) of *S. aureus* cultured overnight was inoculated into 10 ml fresh TSB, and then grown at 37°C with shaking. When the optical density reached 0.5 at 660 nm, various concentrations of nukacin ISK-1or nisin A were added to the medium. After the appropriate incubation, bacterial cells were collected. Total RNA was extracted from the bacterial cells with a FastRNA Pro Blue kit (MP Biomedicals, Solon, OH, USA) in accordance with the manufacturer’s protocol. A 1-µg aliquot of total RNA was reverse-transcribed to cDNA using a first-strand cDNA synthesis kit (Roche, Tokyo, Japan). Using cDNA as template, quantitative PCR was performed using a LightCycler system (Roche). Primers for *braR*, *vraR*, and *graR* (TCS), as well as for *braA, vraD*, and *vraF* (ABC transporter) were constructed and used to determine the optimal conditions for analysis of their expression, and *gyrA* was used as an internal control. Three independent experiments were performed, and the mean was calculated. Statistical analysis was performed with Dunnett’s method. The primers are listed in Table S1.

### Co-culture of *S. aureus* with *S. warneri* or *L. lactis*

The method for the co-culture experiment is summarized schematically in Figure S1. Overnight cultures of *S. aureus* MM30 (MW2 harboring pCL8 [33]), *S. warneri* ISK-1, *S. warneri* ISK-1^-^, *L. lactis* ATCC 11454, and *L. lactis* NZ9000 were adjusted to OD_660_ = 1.0 and diluted ten-fold. Next, 100 µl of bacterial culture (*S. aureus* [10^7^, 10^6^, 10^5^, 10^4^ cells] and *S. warneri* [10^7^ cells], *S. aureus* [10^7^, 10^6^, 10^5^, 10^4^ cells], and *L. lactis* [10^7^ cells]) was mixed well. A 20-µl aliquot of the mixed culture was spotted on a 50% trypticase soy agar (TSA) plates. After overnight incubation at 37°C, the bacterial colonies growing on the agar plate were scraped and suspended in 1 ml of TSB. The appropriate dilutions were plated on TSA and TSA containing chloramphenicol (for selection of *S. aureus*). After 1 day, the colony-forming units (CFUs) grown on TSA and TSA containing antibiotics were determined, and we calculated the percent population of the *S. aureus* strain. We also extracted total RNA from scraped cells and performed cDNA synthesis using the method described above; gene expression analysis was conducted by quantitative PCR. The statistical analysis was conducted by Dunnett’s method for the percentage ratio of the *S. aureus* population and the expression of *braA* and *vraD*.

Next, the co-culture of *S. aureus* TCS or ABC transporter mutants with *S. warneri* or *L. lactis* was investigated using the method described above. The concentrations of bacterial cells used in this assay were 10^7^ cells/ml *S. aureus* mutant and 10^8^ cells/ml *S. warneri* or 10^6^ cells/ml *S. aureus* mutant and 10^8^ cells/ml *L. lactis*. For the co-culture of *S. aureus* with *S. warneri*, TSA containing chloramphenicol (wild-type *S. aureus*, 10 µg/ml) and tetracycline (*S. aureus* mutants, 10 µg/ml) were used for *S. aureus* selection.

### Susceptibility of Bacitracin-treated *S. aureus* to Nukacin ISK-1 and Nisin A

To investigate whether the VraDE expression level affects susceptibility to nisin A and nukacin ISK-1, we evaluated the susceptibility of bacitracin-pretreated *S. aureus* to nisin A and nukacin ISK-1 using the MIC method, as described above, and the spot-on-lawn method described elsewhere [11]. Previously, we reported that bacitracin at a sub-MIC induced VraDE expression significantly [23]. *S. aureus* pretreated with or without bacitracin was used for both methods. *S. aureus* cells (10^9^/ml) were exposed to a sub-MIC (1/8 MIC: 8 µg/ml) of bacitracin (Sigma-Aldrich, Tokyo, Japan) for 30 min. In the spot-on-lawn method, 5 µl of nisin A (6.4 µg/ml) or nukacin ISK-1 (64 µg/ml) were spotted on a double-layered agar plate containing 8 ml of TSA soft agar with 10^6^/ml *S. aureus* cells as the upper layer and 10 ml of TSB agar (1.5%) as the bottom layer. After overnight incubation at 37°C, the diameter of the inhibition zone for bacterial growth was measured in three directions.

Additionally, we constructed the VraDE overexpression strain in the *graRS* mutant and investigated the susceptibility to nisin A and nukacin ISK-1. The gene coding *vraDE* was amplified with specific primers, and then the DNA fragment was cloned into pCL15, which harbored an *E. coli–S. aureus* shuttle vector with the P*spac* promoter [34]. The expression of the cloned gene in pCL15 was significantly induced by the addition of 1 mM isopropyl β-D-1-thiogalactopyranoside (IPTG). The obtained plasmid was electroporated to *S. aureus* RN4220, and then the plasmid was transduced to the *gra* mutant using phage 80 alpha with the method described in a previous study [35]. Plasmids and primers used in the present study are listed in Table S1. Using this strain, susceptibility was investigated by the MIC method and the spot-on-lawn method, as described above.

## Results

### Susceptibility of TCS-inactivated Mutants to nisin A and Nukacin ISK-1

We determined the susceptibility of TCS mutants to nukacin ISK-1 using the MIC and the direct method (Table 3, Figure S2). The *graRS* and *braRS* mutants exhibited higher susceptibility to nukacin ISK-1, and the *vraSR* mutant exhibited higher susceptibility to nukacin ISK-1. The susceptibilities of other TCS mutants other than *braRS*, *graRS* and *vraSR* to nukacin ISK-1 did not change (data not shown). We also the evaluated the susceptibility of TCS mutants to nisin A and nukacin ISK-1 using the direct method (Figure S2), and obtained similar results to those obtained using the MIC method. Furthermore, we determined the susceptibility of the mutants and their complemented strains. We found that each complemented strain could restore the respective mutation (Table 3). Also, we investigated the susceptibility of TCS mutants against nisin A, and found similar results to nukacin ISK-1 except that the susceptibility of the *vraRS* mutant to nisin A did not increase (Table 3, Figure S2).

**Table 3:** Susceptibility to nisin A, nukacin ISK-1 and bacitracin of *S. aureus* mutants.

Strain	Relevant Genotype	MIC (mg/L)		
		Nisin A	Nukacin ISK-1	Bacitracin
MW2	Wild-type	6.4	64	64
FK77	Δ*braRS*	3.2	16	32
MM08	Δ*braS*	3.2	16	32
MM09	*braS* complement in Δ*braS*	6.4	64	64
FK64	Δ*graRS*	3.2	16	64
MM12	*graR* complement in Δ*graRS*	6.4	64	64
FK72	Δ*vraSR*	6.4	32	32
MM231	*vraSR* complement in Δ*vraSR*	6.4	64	64
MM02	Δ*braAB*	3.2	16	32
MM07	Δ*braB*	3.2	16	32
MM10	*braB* complement in Δ*braB*	6.4	64	64
MM03	Δ*vraDE*	3.2	8	16
MM11	*vraDE* complement in Δ*vraDE*	6.4	64	64
MM01	Δ*vraFG*	3.2	32	64
MM17	*vraFG* complement in Δ*vraFG*	6.4	64	64

### Susceptibility of ABC Transporter-inactivated Mutants to nisin A and nukacin ISK-1

Previously, we demonstrated that BraRS regulates two transporters (VraDE and BraAB) for resistance against bacitracin, and that GraRS regulates one transporter (VraFG) [19], [23]. We evaluated the susceptibility of these three mutants (*vraDE, braAB*, and *vraFG* mutants) to nukacin-ISK-1 (Table 3, Figure S2). The *vraFG*, *vraDE*, and *braAB* mutants were more susceptible to nukacin ISK-1. Additionally, we found that each complemented strain could restore the respective mutation (Table 3). Also, we investigated the susceptibilities of these mutants to nisin A, and obtained similar results to those for nukacin ISK-1 (Table 3, Figure S2).

### Effect of Nukacin ISK-1 and Nisin A on the Expression of TCSs and Transporters

Given that the susceptibility to nisin A and nukacin ISK-1 was changed by inactivation of three TCSs (*braRS*, *graRS*, and *vraSR*) and three transporters (*vraFG*, *vraDE*, and *braAB*), we investigated the expressions of those TCSs and transporters upon exposure of bacterial cells to nukacin ISK-1 and nisin A.

**Figure 1:**
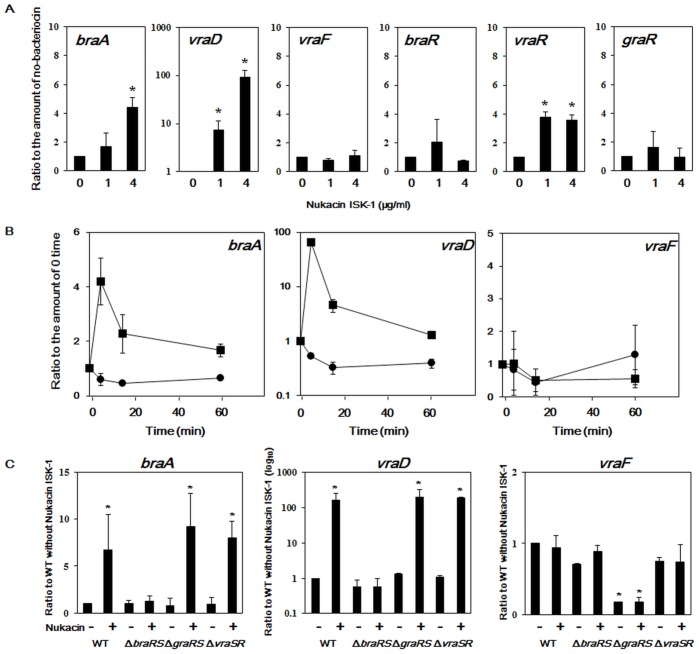
Expression of TCSs and ABC transporters in *S. aureus* exposed to nukacin ISK-1. Analysis of expression levels of *braR*, *graR*, *vraR*, *braA*, *vraD*, and *vraF* were performed as described in the Materials and Methods. (A) *braA*, *vraF, vraD*, *braR*, *graR*, and *vraR* expression in *S. aureus* MW2 exposed to various concentrations of nukacin ISK-1 (5-min exposure). *, statistically significant difference from the wild-type as tested using Dunnett’s method (*p*<0.05). (B) Time course experiment of *braA*, *vraF*, and *vraD* expression in *S. aureus* MW2 exposed to nukacin ISK-1 (4 µg/ml). (C) *braA*, *vraF,* and *vraD* expression in *S. aureus* MW2 and three mutants (*braRS*, *graRS*, and *vraSR* mutant) exposed to nukacin ISK-1 (4 µg/ml). *, statistically significant difference from the wild-type as tested using Dunnett’s method (*p*<0.05).

Among the three TCSs, the expression levels of the *braR* and *graR* transcripts did not increase upon exposure to nukacin ISK-1, whereas the expression of *vraR* was induced (Figure 1A). Regarding the three ABC transporters, the expression of *braA* and *vraD* in the wild-type MW2 strain was rapidly induced by the addition of nukacin ISK-1 to the medium, whereas *vraF* expression was not (Figures 1A and 1B). This induction occurred after 5 min of exposure, after which the transcript levels of both transporters gradually decreased (Figure 1B). The expressions of both *braA* and *vraD* were dose-dependent (Figure 1A). However, the induction of *vraD* and *braA* expression by nukacin ISK-1 was not observed in the *braRS* mutant, although both genes showed increased expression in the *graRS* and *vraSR* mutants (Figure 1C). In the *graRS* mutant, *vraF* expression decreased irrespective of nukacin ISK-1 addition, but in the other two mutants, the *vraF* expression level was not significantly different from that of the wild-type (Figure 1C).

In addition, we investigated the expression of TCSs and transporters in the wild-type and its mutants by addition of nisin A. We obtained results similar to those of nukacin ISK-1, except that nisin A did not induce *vraSR* expression (Figure S3).

Based on these results, we concluded that the expression of two ABC transporters, *vraD* and *braA*, was induced by nukacin ISK-1 and nisin A, and that this effect was mediated by one TCS, BraRS. Also, the expression of another transporter, *vraF*, was not induced, but *vraF* expression was regulated by GraRS.

### Co-culture of *S. aureus* with *S. warneri* or *L. lactis*

**Figure 2:**
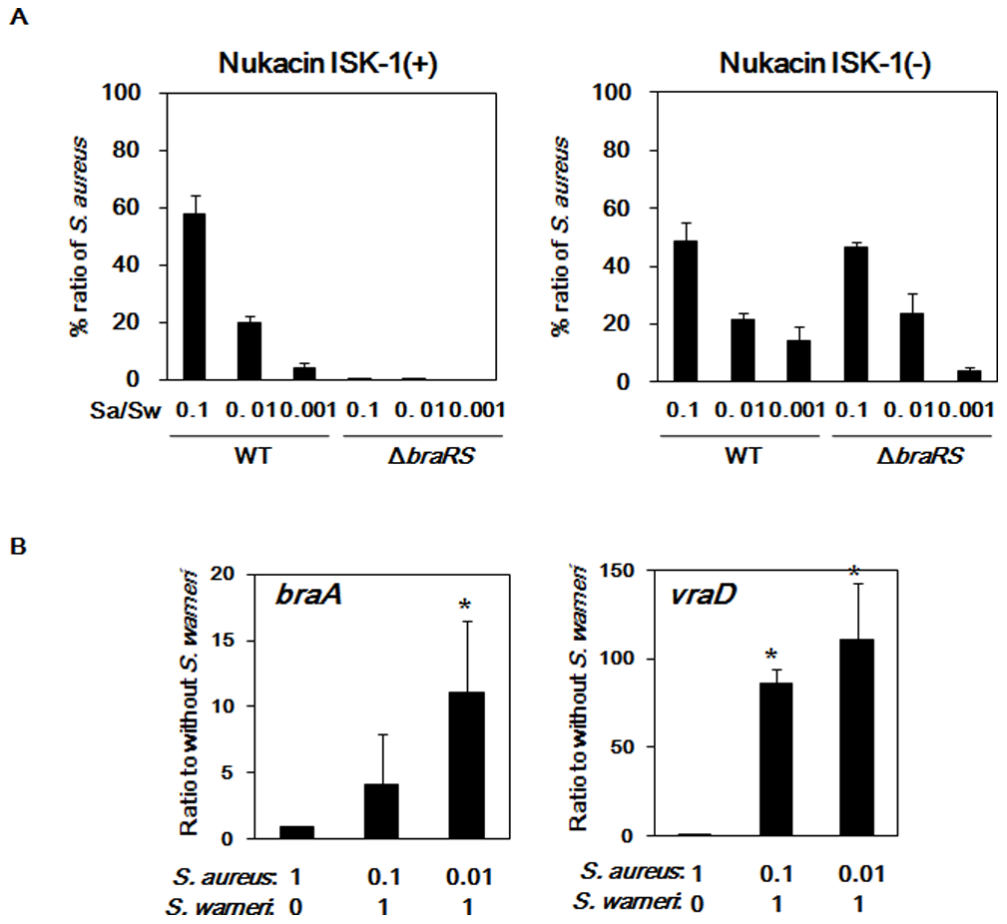
Co-culture of *S. aureus* with *S. warneri* The co-culture experiment was performed with the method described in the Materials and Methods. (A) Percent ratio of the *S. aureus* population when mixed with various concentrations of *S. warneri* ISK-1 and nukacin-non-producing *S. warneri*. (B) Expression of the ABC transporters (*braA* and *vraD*) when mixed with various concentrations of *S. warneri* ISK-1. *p<0.05, as determined by Dunnett’s method for the expression of the ABC transporters (*braA* and *vraD*).

Co-cultures of *S. aureus* MM30 with *S. warneri* ISK-1, *S. warneri* ISK-1ΔpPI-1 (pPI-1 plasmid cured), *L. lactis* ATCC 11454 (nisin A-producing strain), and *L. lactis* NZ9000 (nisin A non-producing strain) were analyzed. When *S. aureus* MM30 was co-cultured with *S. warneri* ISK-1, which produces nukacin ISK-1, the population of the *braRS* mutant was significantly lower at any *S. aureus*/*S. warneri* ratio, compared to that of the wild-type (MM30) (Figure 2A). When *S. aureus* was co-cultured with *S. warneri* ISK-1ΔpPI-1, which does not produce nukacin ISK-1, the population of the *braRS* mutant was similar to that of the wild-type. We evaluated the expression of *vraD* and *braA* under co-culture conditions (Figure 2B). The expression of both increased when *S. aureus* was co-cultured with *S. warneri* ISK-1 at various ratios. In particular, both increased gradually as the ratio of *S. aureus* to *S. warneri* decreased before spotting on the TSA plate.

*S. aureus* MM30 was co-cultured with *L. lactis* ATCC 11454, which produces nisin A, or NZ9000, which does not. Results were similar to those of nukacin ISK-1 (Figure S4).

**Figure 3:**
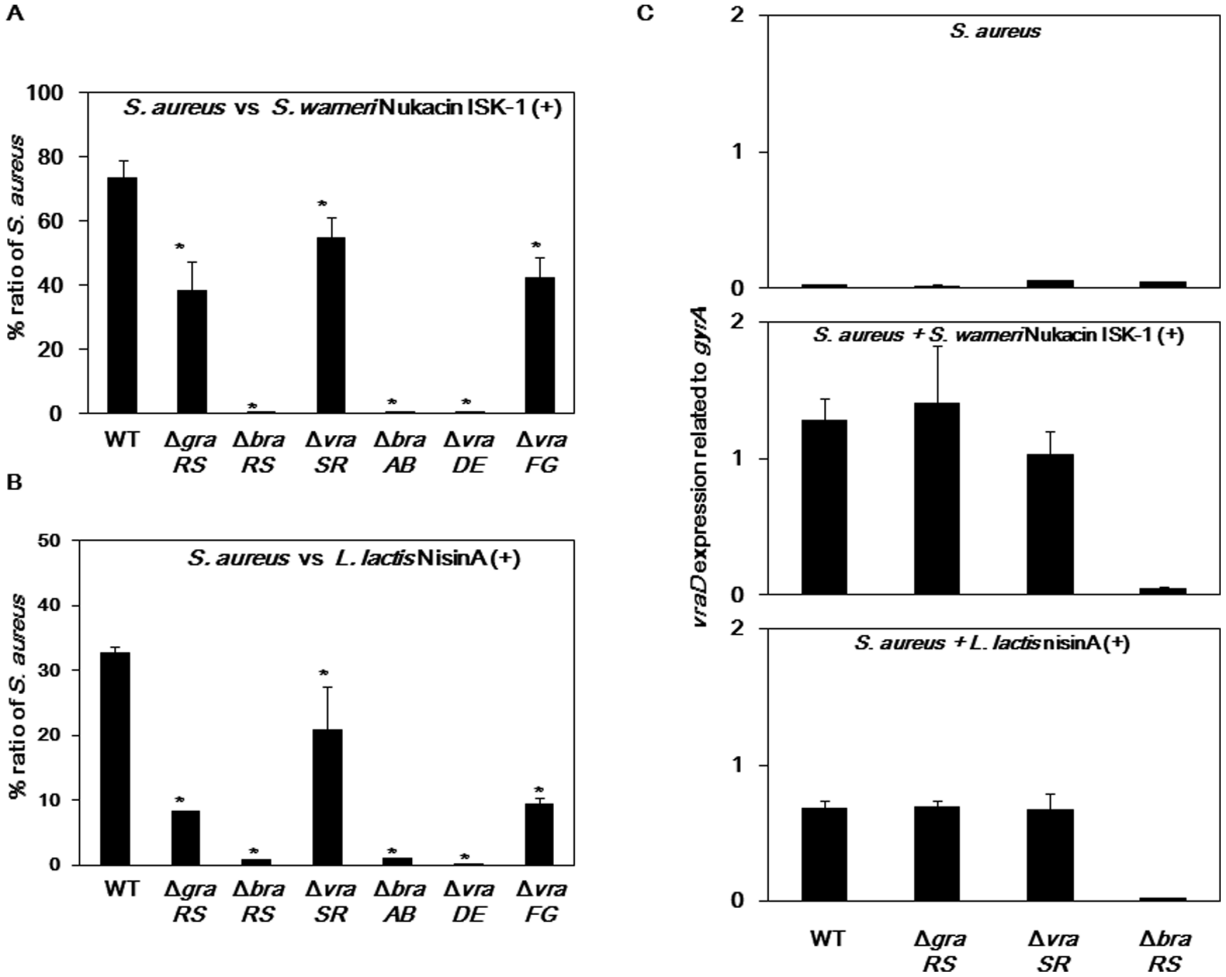
Co-culture of *S. aureus* TCS and transporter mutants with *S. warneri* or *L. lactis*. The co-culture assay is described in the Materials and Methods. A 100-µl aliquot of *S. warneri* ISK-1 (10^8^ cells/ml) (A) or *L. lactis* ATCC 11454 (10^8^ cells/ml) (B) was mixed with 100 µl of *S. aureus* (10^7^ cells/ml for *S. warneri* and 10^6^ cells/ml for *L. lactis*). (C) Expression of the ABC transporter *vraDE* in the mutants when mixed with *S. warneri* ISK-1 or *L. lactis*. *p<0.05, as determined by Dunnett’s method for the percent ratio of the *S. aureus* population and the expression of the ABC transporters.

Figure 3A shows the *S. aureus* population ratio when 10^6^ cells of *S. aureus* mutants were mixed with 10^7^ *S. warneri* ISK-1 cells. The *braRS, braAB*, and *vraDE* mutants exhibited drastically decreased population ratios compared with that of the wild-type. In addition, the *graRS, vraSR*, and *vraFG* mutants showed slight decreases compared with the wild-type.

Similar results were obtained when 10^5^ cells of *S. aureus* mutants were mixed with 10^7^ *L. lactis* cells (Figure 3B). When co-cultured with the ATCC 11454 strain, the population ratios of the *braRS*, *braAB,* and *vraDE* mutants were significantly lowered, compared to that of the wild-type. Additionally, we investigated *vraD* expression in TCS mutants. The wild-type, *graRS* and *vraSR* mutants showed significantly increased *vraD* expression upon co-culture with bacteriocin-producing strains (Figure 3C). However, *vraD* expression did not increase in the wild-type, *graRS* and *vraSR* mutants when co-cultured with bacteriocin-non-producing strains (data not shown).

From the results of co-culture assay, we found that the inactivation of one TCS (*braRS*) and two BraRS-regulated transporters (*braAB* and *vraDE*) caused a significant decrease in the *S. aureus* population when co-cultured with a nukacin ISK-1- or nisin A-producing strain.

### Susceptibility of Bacitracin-treated *S. aureus* to nisin A and nukacin ISK-1

Because *vraDE* expression of the *S. aureus* wild-type was induced by nisinA or nukacin ISK-1, we investigated whether the VraDE-overexpressing strain showed higher resistance to nisinA and nukacin ISK-1. We used *S. aureus* pretreated with a sub-MIC of bacitracin, which induced the expression of VraDE [23] but was not bactericidal. The wild-type strain pretreated with bacitracin showed an increased nisin A and nukacin ISK-1 MICs compared to that without bacitracin (Table 4). Also, the MICs of the *graRS* and *vraRS* mutants against nisin A and nukacin ISK-1 were increased by pretreatment with bacitracin, whereas the MIC of the *braRS* mutant did not change. In addition, we constructed a VraDE-overexpression strain using the pCL15 plasmid. VraDE overexpression in the *graRS* mutant caused decreased susceptibility to both nisin A and nukacin ISK-1 (Table 4).

**Table 4:** Susceptibility of bacitracin-treated and *vraDE*-overexpressing *S. aureus* to nisin A and nukacin ISK-1.

		MIC (mg/L)			
Strain	Relevant Genotype	Bacitracin (−)		Bacitracin (+)	
		Nisin A	Nukacin ISK-1	Nisin A	Nukacin ISK-1
MW2	Wild-type	6.4	64	12.8	128
FK64	Δ*apsRS* in MW2	3.2	32	12.8	128
FK72	Δ*vraSR* in MW2	6.4	32	12.8	128
FK77	Δ*braRS* in MW2	3.2	16	3.2	16
		**IPTG (–)**		**IPTG (+)**	
**Strain**	**Relevant Genotype**	**Nisin A**	**Nukacin ISK-1**	**Nisin A**	**Nukacin ISK-1**
MM31	*vraDE* in FK64	3.2	32	12.8	128

Expression of *braR, graR, vraR, braA, vraD*, and *vraF* was determined using the method described in the Materials and Methods. (A) *braA*, *vraF, vraD*, *braR*, *graR*, and *vraR* expression in *S. aureus* MW2 exposed to various concentrations of nisin A (5 min exposure). *, statistically significant difference from the wild-type as tested using Dunnett’s method (*p*<0.05). (B) Time course experiment of *braA*, *vraF*, and *vraD* expression in *S. aureus* MW2 exposed to nisin A (16 µg/ml). (C) *braA*, *vraF*, and *vraD* expression in *S. aureus* MW2 and three mutants (*braRS*, *graRS*, and *vraSR* mutant) exposed to nisin A (16 µg/ml). *, statistically significant difference from the wild-type as tested using Dunnett’s method (*p*<0.05).

## Discussion

We performed a comprehensive analysis of the TCSs involved in the susceptibility to the class I bacteriocin, nukacin ISK-1, in *S. aureus* and identified several TCSs to be associated with nukacin ISK-1 susceptibility (Figure S2, Table 3). Previously, BraRS and GraRS were also shown to be associated with bacitracin and nisin A resistance [23], [24], [25], [36]. In addition, Hiron *et al*. demonstrated that BraRS is activated by nisin A, which induces the expression of transporters [24], as confirmed by the results in this study (Figure 1, Figure S3). Therefore, BraRS is involved in the resistance to nukacin ISK-1, nisin A and bacitracin. Bacitracin binds to undecaprenyl pyrophosphate, resulting in inhibition of lipid II formation, whereas nisin A and nukacin ISK-1 bind to lipid II [9], [10]. Vancomycin also binds to the D-alanine-D-alanine molecule in lipid II; however, the *braRS* mutant did not exhibit marked susceptibility to vancomycin [18]. Nisin A binds to the pyrophosphate moiety of lipid II, resulting in pore formation in the membrane [9]. Therefore, we propose that BraRS is associated with susceptibility to antibacterial agents related to the membrane-anchoring region of lipid II. Recently, BraAB was found to act as a cofactor for BraRS but not as a direct resistance factor, suggesting that it is associated with the regulation of *vraDE* [24]. BraRS in *S. aureus* shows homology with TCSs of other gram-positive bacteria, including *Enterococcus*, *Bacillus* and *Streptococcus* [37], [38], [39], [40]. Therefore, the BraRS system is widely conserved in gram-positive bacteria.

GraRS is involved in susceptibility to cationic peptides such as defensins, gentamicin, and vancomycin [41], [42], [43] because it regulates two factors, *dlt* and *mprF* (*fmtC*), both of which influence the cell surface charge of bacteria [36], [42], [43], [44]. Inactivation of *graRS* causes an increase in the negative charge of the cell surface, resulting in increased attraction of the cationic peptides nisin A and nukacin ISK-1, but not bacitracin, to the cell membrane. In addition, *vraFG* (downstream of *graRS*) is associated with susceptibility to nisin A and nukacin ISK-1, but not bacitracin. Recently, Falord *et al*. demonstrated that VraFG did not act as a detoxification module but was associated with GraRS activation [45]. Therefore, the difference in susceptibilities between nisin A/nukacin ISK-1 and bacitracin may be due to the charges of these peptides.

VraSR (vancomycin resistance associated sensor/regulator) was first identified as a factor responsible for vancomycin susceptibility [46]. Further investigations revealed that this TCS regulates many factors involved in cell wall biosynthesis and that it is associated with susceptibility to cell wall synthesis inhibitors including beta-lactams, cycloserine, teicoplanin, and bacitracin [47], [48], [49]. In this study, the inactivation of *vraSR* led to an increase in susceptibility to nukacin ISK-1, but not nisin A (Table 3 and Figure S2). Moreover, nukacin ISK-1 induced *vraSR* expression (Figure 1A), whereas nisin A did not (Figure S3). Although nisin A also exhibits an inhibitory effect on cell wall biosynthesis, we did not detect elevated *vraSR* expression under our conditions. Nukacin ISK-1 and nisin A are type A lantibiotics, but their subtypes (type A [I] and type A [II], respectively) and structure differ (Figure S5). Also, their modes of action differ; nisin A exhibits a bactericidal effect by causing pore formation and the inhibition of cell wall biosynthesis [7], [8], whereas nukacin ISK-1 acts as a bacteriostatic agent by inhibiting cell wall biosynthesis [11]. Therefore, we hypothesize that the different modes of action of these two bacteriocins reflect the different responses in terms of *vraSR* expression.

We evaluated the competition between two bacterial strains using a co-culture method. Notably, the inactivation of three TCSs, but especially *graRS* and *braRS*, caused significantly increased susceptibility to nisin A and nukacin ISK-1 by the direct and the MIC methods (Figure S2 and Table 3); however, only one TCS, BraRS, was a major contributor to *S. aureus* survival in co-culture with *S. warneri or L. lacti,* which produces bacteriocin (Figures 2, 3 and S4). In particular, the *graRS* mutant showed different results between the direct and co-culture methods. We hypothesized that this difference in the *graRS* mutant was due to the different level of VraDE expression upon exposure of the mutant to nisin A and nukacin ISK-1. In the direct assay, *S. aureus* cells that expressed VraDE at a very low level were exposed to relatively high concentrations of nisin A and nukacin ISK-1. In the early period after bacteriocin exposure, VraDE expression was not sufficient for BraRS-mediated nisin A/nukacin ISK-1 resistance; thus the *graRS* mutation, which exhibited a more negatively charged cell surface [42], [43], showed marked susceptibility to nisin A and nukacin ISK-1. Conversely, *S. aureus* cells in the co-culture are exposed to a low concentrations (non-lethal) of nisin A or nukacin ISK-1 during the early stage of co-culture, and so VraDE expression is induced. Upon exposure to a high concentration of nisin A or nukacin ISK-1 during further incubation, *S. aureus* expressed VraDE at a level sufficient for resistance. Table 4 reflects our hypothesis that pretreatment of the *graRS* mutant with bacitracin resulted in marked nisin A and nukacin ISK-1 resistance. Additionally, we obtained the same result when *S. aureus* cells were pretreated with nisin A (data not shown). Furthermore, similar results were obtained using the VraDE overexpression strain (Table 4). Therefore, the different results of the direct and co-culture methods using the *graRS* mutant were due to the different VraDE expression levels upon exposure of *S. aureus* to a high concentration of nisin A or nukacin ISK-1.

Based on our findings, we propose that BraRS and GraRS have distinct functions in terms of resistance to nisin A and nukacin ISK-1. BraRS is an intrinsic factor for such resistance. However, upon exposure of *S. aureus* to a relatively high level of nisin A or nukacin ISK-1, GraRS is important for resistance until significant induction of VraDE expression by BraRS occurs. Therefore, these two TCSs function coordinately in resistance to nisin A and nukacin ISK-1. Furthermore, in addition to these two TCSs, VraSR is independently activated upon inhibition of cell wall biosynthesis. Class I bacteriocins, such as nisin A and nukacin ISK-1 inhibit cell wall biosynthesis, although increased expression of VraSR was identified only in *S. aureus* cells exposed to nukacin ISK-1. Our results strongly indicate that *S. aureus* possesses three distinct class-I-bacteriocin-resistance systems.

**Figure 4:**
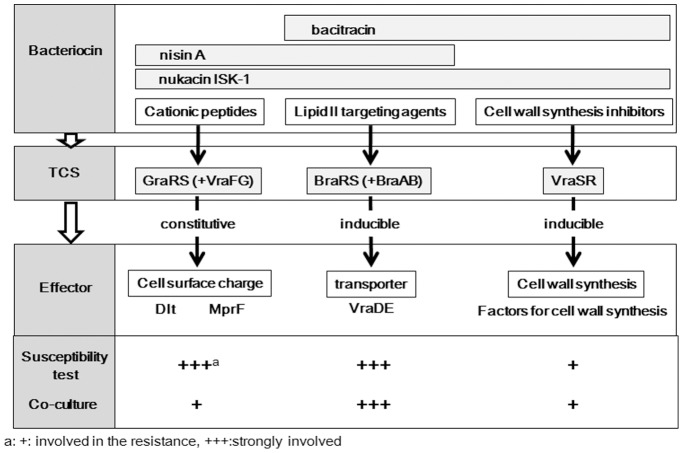
Association of TCSs and ABC transporters with susceptibility to class I bacteriocins, nisin A and nukacin ISK-1.

In conclusion, we demonstrated that several TCSs and ABC transporters in *S. aureus* are associated with resistance to bacteriocins produced by other bacteria. Notably, *S. aureus* possesses multiple TCSs that resist nisin A and nukacin ISK-1 (Figure 4). In particular, the BraRS system is specific for nisin A and nukacin ISK-1. Conversely, GraRS and VraSR confer broad-spectrum resistance against cationic peptides and cell-wall synthesis inhibitors, respectively. Our findings suggest that *S. aureus* possesses several TCSs that facilitate its survival in complex bacterial communities.

## Supporting Information

Figure S1 **Method for the co-culture experiment.**
(TIF)

Figure S2 **Susceptibility of TCS- and ABC transporter-inactivated mutants to nukacin ISK-1 and nisin A.** The susceptibilities of *S. aureus* MW2 and its TCS- or ABC transporter-mutants to nukacin ISK-1 and nisin A were evaluated by the direct method. (A) In total, 2 µl of overnight cultures of bacteriocin-producing strains were spotted on an MRS agar plate. After overnight incubation at 37°C, pre-warmed MRS soft agar (0.75%) containing *S. aureus* was poured over the surface of the MRS agar plate. Plates were incubated for 20 h at 37°C. (B) The diameters of the inhibition zones surrounding the bacteriocin-producing strain were measured in three directions. Three experiments were performed independently, and the average result of the three experiments was calculated. *, statistically significant difference from the wild-type as tested using Dunnett’s method (*p*<0.05). The error bar represents the standard deviation.
(TIF)

Figure S3 **Expression of TCSs and ABC transporters in** ***S. aureus*** **exposed to nisin A.**
(TIF)

Figure S4 **Co-culture of** ***S. aureus*** **with** ***L. lactis.*** Co-culture experiment was performed as described in the Materials and Methods. (A) Percent ratio of the *S. aureus* population when mixed with various concentrations of *L. lactis* ATCC 11454 and nisin A-non-producing *L. lactis* NZ9000. (B) Expression of ABC transporters (*braA* and *vraD*) when mixed with various concentrations of *L. lactis* ATCC 11454. *p<0.05, as determined by Dunnett’s method for expression of the ABC transporters (*braA* and *vraD*).
(TIF)

Figure S5 **Structures of nisin A and nukacin ISK-1.** (A) nisin A; (B) nukacin ISK-1. Shaded residues indicate amino acids: A-S-A, lanthionine; Abu-S-A, 3-methyllanthionine; Dha, dehydroalanine; Dhb, dehydrobutyrine; fM, *N*-formylmethionine.
(TIF)

Table S1 (DOCX)
