# Supplementary figures and images for: Three Distinct Two-Component Systems Are Involved in Resistance to the Class I Bacteriocins, Nukacin ISK-1 and Nisin A, in Staphylococcus aureus
Source: PLoS One. 2013 Jul 22;8(7):e69455. doi: 10.1371/journal.pone.0069455 (PMC3718698; doi:10.1371/journal.pone.0069455)

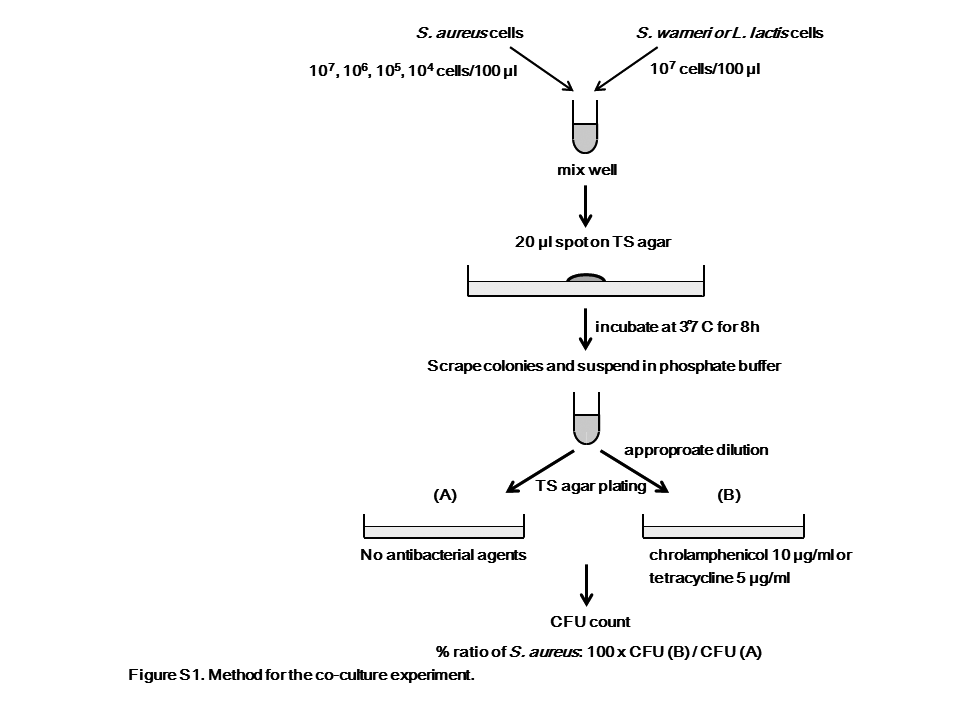

Supplement: Figure S1 — Method for the co-culture experiment. (TIF) [file pone.0069455.s001.tif]

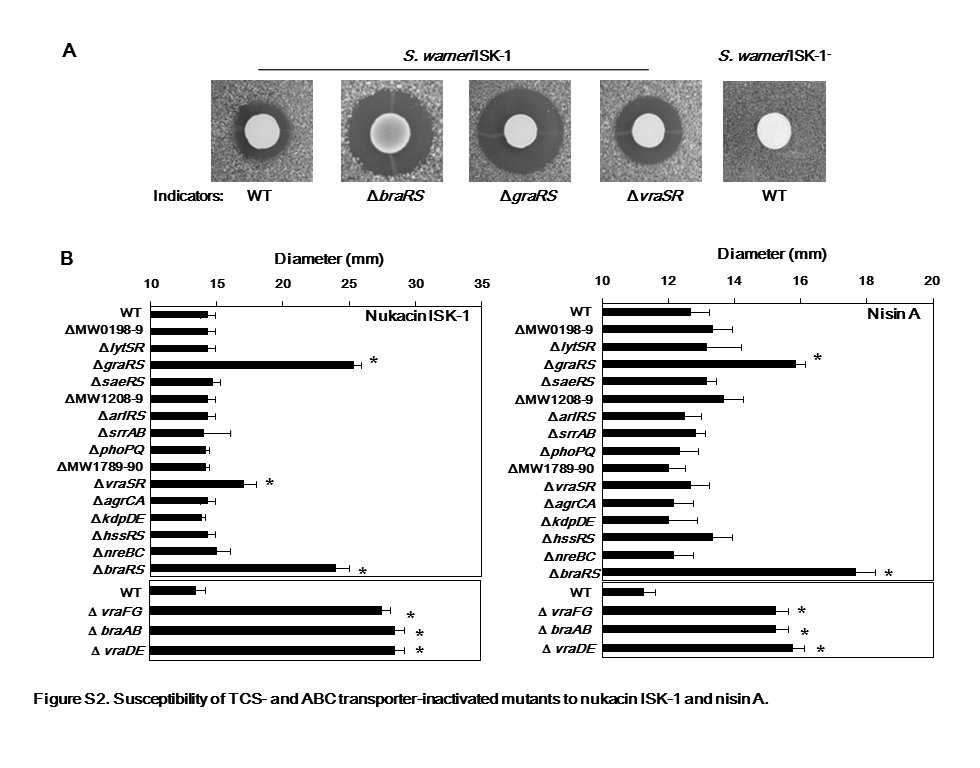

Supplement: Figure S2 — Susceptibility of TCS- and ABC transporter-inactivated mutants to nukacin ISK-1 and nisin A. The susceptibilities of S. aureus MW2 and its TCS- or ABC transporter-mutants to nukacin ISK-1 and nisin A were evaluated by the direct method. (A) In total, 2 µl of overnight cultures of bacteriocin-producing strains were spotted on an MRS agar plate. After overnight incubation at 37°C, pre-warmed MRS soft agar (0.75%) containing S. aureus was poured over the surface of the MRS agar plate. Plates were incubated for 20 h at 37°C. (B) The diameters of the inhibition zones surrounding the bacteriocin-producing strain were measured in three directions. Three experiments were performed independently, and the average result of the three experiments was calculated. *, statistically significant difference from the wild-type as tested using Dunnett’s method (p<0.05). The error bar represents the standard deviation. (TIF) [file pone.0069455.s002.tif]

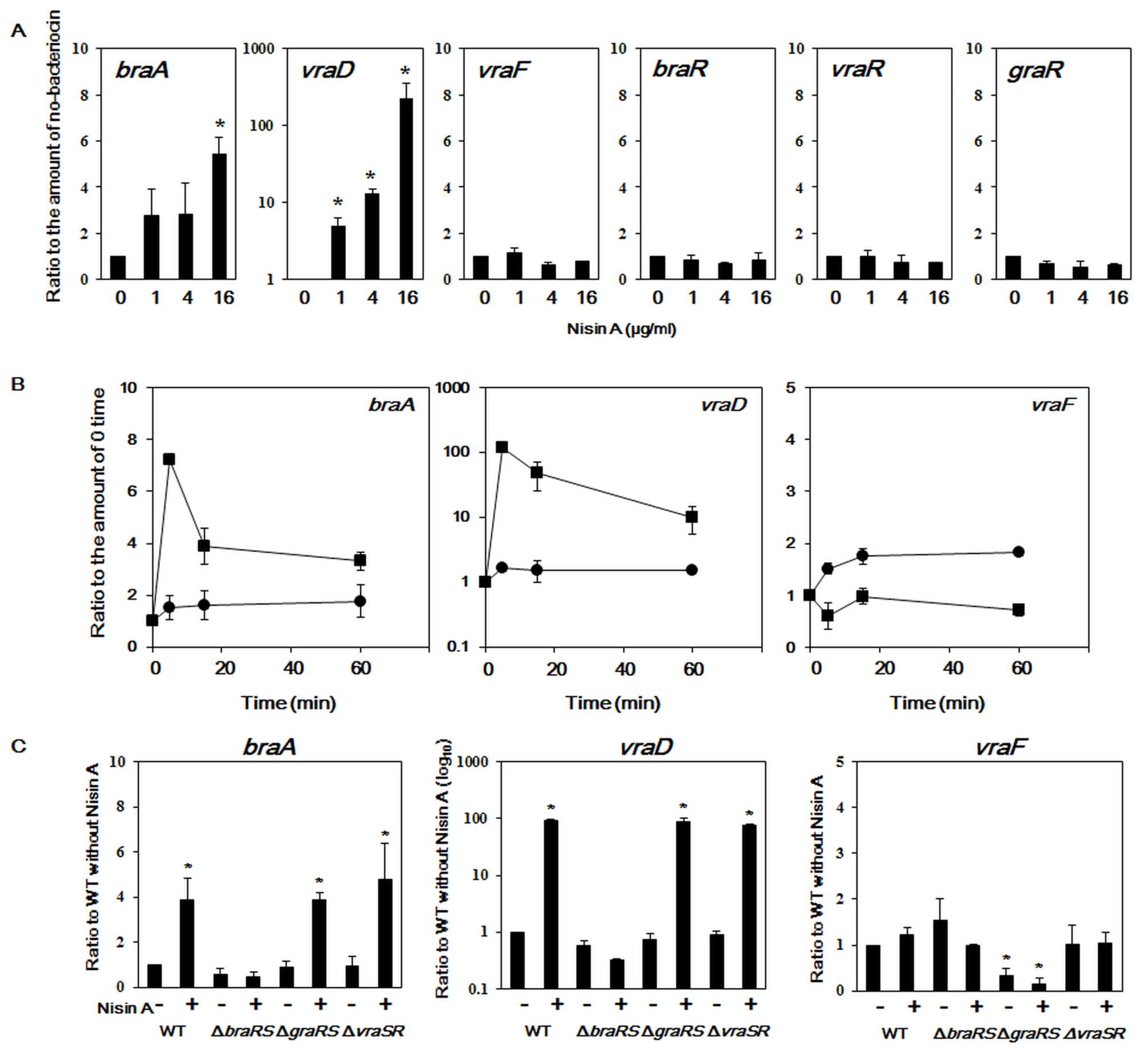

Supplement: Figure S3 — Expression of TCSs and ABC transporters in S. aureus exposed to nisin A. (TIF) [file pone.0069455.s003.tif]

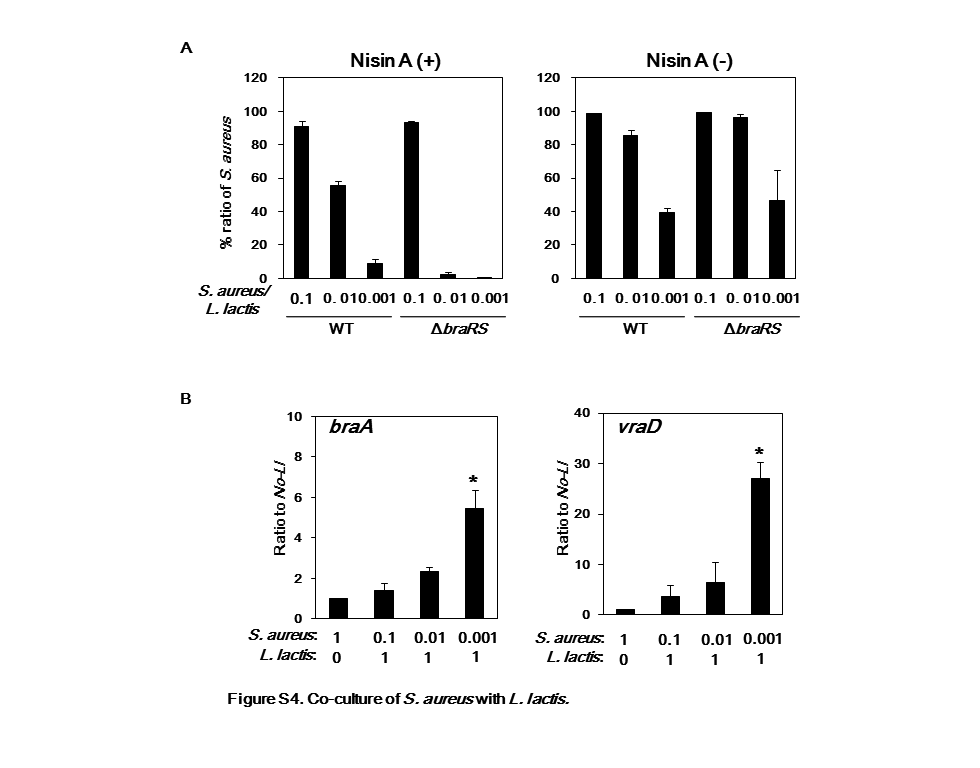

Supplement: Figure S4 — Co-culture of S. aureus with L. lactis. Co-culture experiment was performed as described in the Materials and Methods. (A) Percent ratio of the S. aureus population when mixed with various concentrations of L. lactis ATCC 11454 and nisin A-non-producing L. lactis NZ9000. (B) Expression of ABC transporters (braA and vraD) when mixed with various concentrations of L. lactis ATCC 11454. *p<0.05, as determined by Dunnett’s method for expression of the ABC transporters (braA and vraD). (TIF) [file pone.0069455.s004.tif]

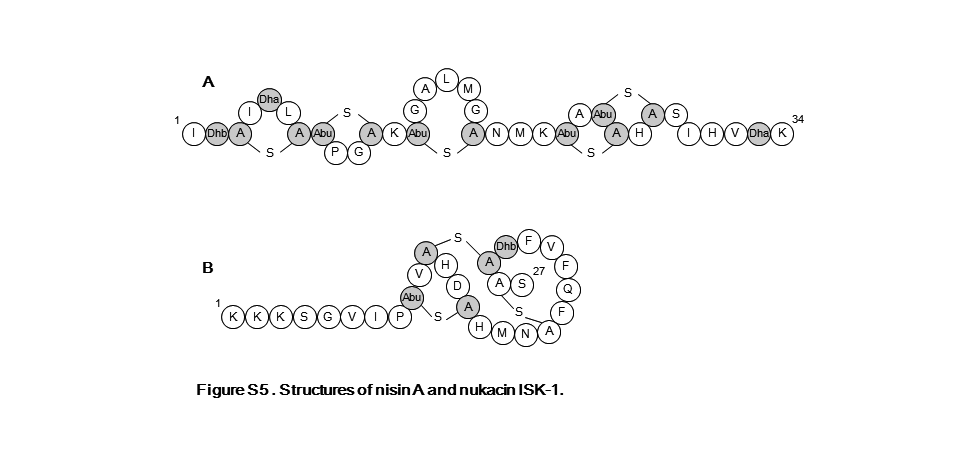

Supplement: Figure S5 — Structures of nisin A and nukacin ISK-1. (A) nisin A; (B) nukacin ISK-1. Shaded residues indicate amino acids: A-S-A, lanthionine; Abu-S-A, 3-methyllanthionine; Dha, dehydroalanine; Dhb, dehydrobutyrine; fM, N-formylmethionine. (TIF) [file pone.0069455.s005.tif]
