# Supplementary material for: Three Distinct Two-Component Systems Are Involved in Resistance to the Class I Bacteriocins, Nukacin ISK-1 and Nisin A, in Staphylococcus aureus
Source: PLoS One. 2013 Jul 22;8(7):e69455. doi: 10.1371/journal.pone.0069455 (PMC3718698; doi:10.1371/journal.pone.0069455)
Supplement: Table S1 — (DOCX) [file pone.0069455.s006.docx]

**Supplemental Figure Legends**

**Figure S1. Method for the co-culture experiment.**

**Figure S2. Susceptibility of TCS- and ABC transporter-inactivated mutants to nukacin ISK-1 and nisin A.**

The susceptibilities of *S. aureus* MW2 and its TCS- or ABC transporter-mutants to nukacin ISK-1 and nisin A were evaluated by the direct method. (A) In total, 2 µl of overnight cultures of bacteriocin-producing strains were spotted on an MRS agar plate. After overnight incubation at 37°C, pre-warmed MRS soft agar (0.75%) containing *S. aureus* was poured over the surface of the MRS agar plate. Plates were incubated for 20 h at 37°C. (B) The diameters of the inhibition zones surrounding the bacteriocin-producing strain were measured in three directions. Three experiments were performed independently, and the average result of the three experiments was calculated. *, statistically significant difference from the wild-type as tested using Dunnett’s method (*p* < 0.05). The error bar represents the standard deviation.

**Figure S3. Expression of TCSs and ABC transporters in *S. aureus* exposed to nisin A.**

Expression of *braR, graR, vraR, braA, vraD*, and *vraF* was determined using the method described in the Materials and Methods. (A) *braA*, *vraF, vraD*, *braR*, *graR*, and *vraR* expression in *S. aureus* MW2 exposed to various concentrations of nisin A (5 min exposure). *, statistically significant difference from the wild-type as tested using Dunnett’s method (*p* < 0.05). (B) Time course experiment of *braA*, *vraF*, and *vraD* expression in *S. aureus* MW2 exposed to nisin A (16 µg/ml). (C) *braA*, *vraF*, and *vraD* expression in *S. aureus* MW2 and three mutants (*braRS*, *graRS*, and *vraSR* mutant) exposed to nisin A (16 µg/ml). *, statistically significant difference from the wild-type as tested using Dunnett’s method (*p* < 0.05).

**Figure S4. Co-culture of *S. aureus* with *L. lactis.***

Co-culture experiment was performed as described in the Materials and Methods.

(A) Percent ratio of the *S. aureus* population when mixed with various concentrations of *L. lactis* ATCC 11454 and nisin A-non-producing *L. lactis* NZ9000. (B) Expression of ABC transporters (*braA* and *vraD*) when mixed with various concentrations of *L. lactis* ATCC 11454. **p* < 0.05, as determined by Dunnett’s method for expression of the ABC transporters (*braA* and *vraD*).

**Figure S5. Structures of nisin A and nukacin ISK-1.**

(A) nisin A; (B) nukacin ISK-1. Shaded residues indicate amino acids: A-S-A, lanthionine; Abu-S-A, 3-methyllanthionine; Dha, dehydroalanine; Dhb, dehydrobutyrine; fM, *N*-formylmethionine.

Table S1. Plasmids and primers.

| Plasmid | Characteristics | | |
| --- | --- | --- | --- |
| pCL52.1 | *E. coli – S. aureus* shuttle vector, thermo-sensitive plasmid, Amp^r1^, TC^r2^ (35) | | |
| pCL8 | *E. coli – S. aureus* shuttle vector, Amp^r^, CP^r3^ (33) | | |
| pCL15 | *E. coli – S. aureus* shuttle vector, Amp^r^, CP^r3^ (34) | | |
| pMM01 | PCR fragment (*vraFG*-KO-F+ *vraFG*-KO-R) / pCL52.1 | | |
| pMM17 | PCR fragment (*vraFG*-comp-F+ *vraFG*-comp-R) / pCL8 | | |
| pMM12 | PCR fragment (*graR*-comp-F+ *graR*-comp -R) / pCL8 | | |
| pMM31 | PCR fragment (*vraDE*-comp-F+ *vraDE*-comp-R) / pCL8 | | |
| pMM231 | PCR fragment (*vraSR*-comp-F+ *vraSR*-comp-R) / pCL8 | | |
| Gene ID | Gene | Primer-Forward | Primer-Reverse |
| Quantitative PCR | | | |
| MW0006 | *gyrA* | *5’-aaggtgttcgcttaattcgc-3’* | *5’-attgcatttcctggtgtttc-3’* |
| MW0621 | *apsR/graR* | *5’-ggatcaagtgatgagtatggaa-3’* | *5’-acagcatcttgccaagtca-3’* |
| MW0623 | *vraF* | *5’-caacaggtgcactggatt-3’* | *5’-tcgtcatccccttggtat-3’* |
| MW2543 | *bceA* | *5’-caccttcagttagtccatca-3’* | *5’-gctacgacagcacttaatca-3’* |
| MW2545 | *bceR* | *5’-ttaaccaacatcaacctcag-3’*- | *5’-ccccatttgtattgccat-3’* |
| MW2620 | *vraD* | *5’-cacttgccaaattccgta-3’* | *5’-aatacctaatgctgtcgtga-3’* |
| MW1825 | *vraR* | 5’*-attagatgcaggtgtcgata*-3’ | 5’*-ccatttctcgttctgtaagc*-3’ |
| Primer | Gene | Primer-Forward | Primer-Reverse |
| Construction for gene inactivation | | | |
| *vraFG*-KO | *vraFG* | *5’-caggatccaaggaaggctcacaagtc-3’* | *5’-ataagcttcaaacgttggtccaccta-3’* |
| Construction for complementation | | | |
| *vraFG*-comp | *vraFG* | *5’-aagtcgacgatacaagtgccaaagcc-3’* | *5’-ccggatccgttcgagaatccgaatcc-3’* |
| *graR*-comp | *graR/apsRS* | *5’-cgggatccgatattgggtgatatggat-3’* | *5’-cgaagcttcatttcaaattattcatgag-3’* |
| *vraDE*-comp | *vraDE* | *5’-cgggatccttcgttgcgattatgggg-3’* | *5’-cgaagcttgcatcttttaatcataagtg-3’* |
| *vraSR-comp* | *vraSR* | *5’-aaaagctttcggagacgtagaggtga-3’* | *5’-gtggatccgtgattggcgtaagtaac-3’* |

^1^ Ampicillin resistance.

^2^ Tetracycline resistance.

^3^ Chloramphenicol resistance.
